# Reduced PDE4D7 in prostate cancer correlates with genomic downregulation within the upstream PDE4D coding region

**DOI:** 10.2144/fsoa-2023-0064

**Published:** 2023-07-29

**Authors:** Chloe Gulliver, George S Baillie, Ralf Hoffmann

**Affiliations:** 1 School of Cardiovascular & Metabolic Health, College of Medical, Veterinary & Life Science, University of Glasgow, Glasgow, G12 8QQ, U.K.; 2 Philips Research Europe, High Tech Campus, Eindhoven, 5656AE, The Netherlands

**Keywords:** biomarkers, cancer, diagnostic, genomics, phosphodiesterase, prognostic, prostate, stratification

## Abstract

**Aim:**

*PDE4D7* expression is significantly associated with prostate cancer (PCa) progression, representing an attractive prognostic biomarker. We sought to determine whether other genes in the *PDE4D* coding region were associated.

**Patients & methods:**

RNA from biopsy punch samples of resected tumor tissue was analyzed via RNA sequencing. RT-qPCR was used to determine *PDE4D7* score.

**Results:**

Numerous genomic sequences within the *PDE4D* coding region on Chr5q12 revealed similar mRNA expression profiles to *PDE4D7*. *PART1* had a significantly similar expression pattern to *PDE4D7* across samples, correlating with disease progression. However, many other genes also exhibited matched expression to *PDE4D7*, including miRNAs and lncRNAs.

**Conclusion:**

These novel *PDE4D7*-associated genes, many of which are previously uncharacterized in cancer, represent putative PCa biomarkers and could have mechanistic roles in PCa progression.

## Plain language summary

Identification of biological molecules that can indicate the presence of a disease are known as biomarkers and are valuable in medical research. For prostate cancer, the *PDE4D7* gene is helpful for determining how the disease is getting worse. To understand more, this study explored whether genes located near *PDE4D7* are also connected to prostate cancer. The analysis of tumor biopsies revealed that many of these nearby genes exhibited similar expression levels to *PDE4D7*, indicating their association with disease development. These findings suggest that several of these nearby genes could serve as potential biomarkers for prostate cancer by giving important clues to understand and detect the disease better.

Given the substantial heterogeneity in prostate cancer (PCa) treatment responses to standard therapies vary, therefore a prognostic biomarker which could accurately indicate disease progression would influence personalized treatment decisions and be beneficial in identifying novel therapeutic targets [1]. Dysregulated cyclic AMP (cAMP) signaling is associated with many diseases including cancer, and is particularly implicated in the progression from localised androgen-sensitive (AS) PCa to aggressive castration-resistant PCa (CRPC) [2]. Viable cAMP signalling depends on the compartmentalization of signaling intermediates such as cAMP effector proteins and phosphodiesterases (PDEs) [3]. The PDE4D sub-family is germane in PCa, with the long isoform PDE4D7 being of unique importance [4]. Previously we identified that *PDE4D7* expression inversely correlates with disease progression, with significant downregulation of the isoform observed between localised PCa and metastatic CRPC samples [1,4]. Diminished *PDE4D7* transcript level also correlated with an increased risk of post-surgical biochemical relapse in a patient cohort, identifying its value in risk stratification [5,6]. Additionally, there is a positive association between *PDE4D7* expression and presence of the *TMPRSS2* and ETS transcription factor family member *ERG* (*TMPRSS2-ERG*) gene rearrangement which has been linked to PCa development, leading to the proposition of a combined ‘CAPRA & PDE4D7’ score [5,7]. Interestingly, combining analysis of other long PDE4D isoforms, *PDE4D5* and *PDE4D9*, can be used to further improve the prognostic power of the combination risk model [8]. These studies have highlighted the potential of *PDE4D7* not only as a prognostic biomarker in patient risk stratification to distinguish between insignificant and aggressive PCa tumors, but also the potential of pharmacological enhancement of PDE4D7 activity, representing novel therapeutic avenues for aggressive PCa.

A study by Wedge *et al.* (2018) investigating genomic rearrangements via whole genome sequencing of >100 PCa tissue samples identified losses at chr5:60–100 Mb in *ETS*-negative tumors, as well as homozygous deletions within chr5:55–59 Mb (region encoding *PDE4D*) in *ETS*-positive tumors [9]. Given that exons 1–3 of *PDE4D7* are located between the region of chr5:59–60 Mb, presence of the *TMPRSS2-ERG* gene fusion may influence the variation observed in *PDE4D* isoform expression [6,8]. Given this, and the evident involvement of the *PDE4D* gene in PCa development and progression, we sought out to investigate the genomic profile of the *PDE4D* coding region on chromosome 5 in PCa tissue samples to ascertain differences in other genes within this area in correlation with *PDE4D7* expression.

## Methods

Two independent NGS transcriptomics expression data sets (n = 533 and n = 151) from PCa patient biopsy punch samples of surgically resected tumor tissue were used in this study. The local Institutional Review Boards approved the collection of patient tissue for clinical research. RNA sequencing of tissue extracted RNA and data processing was performed as previously described [8]. Generation of normalized *PDE4D* transcript expression was performed via RT-qPCR, by subtracting the Cq of respective transcripts from the averaged reference gene Cq, then transformed to their respective transcript scores. To investigate the expression of genomic elements on chromosome 5 in the region upstream and downstream of where exons 1–3 of *PDE4D7* are located (approximately chr5:59,000,000 to chr5:60,500,000), we created a heatmap of the expression of respective transcripts together with the level of *PDE4D5, PDE4D7* and *PDE4D9* expression. *PDE4D5, PDE4D7* and *PDE4D9* scores, as well as the transcript per million expression values for the respective transcripts, were transferred into z-scores across all samples. As a result, all genes of the heatmap have a mean expression of 0 and a SD of 1. These z-score transformed expression values were used as input for the expression heatmaps.

## Results

**Figure 1.**
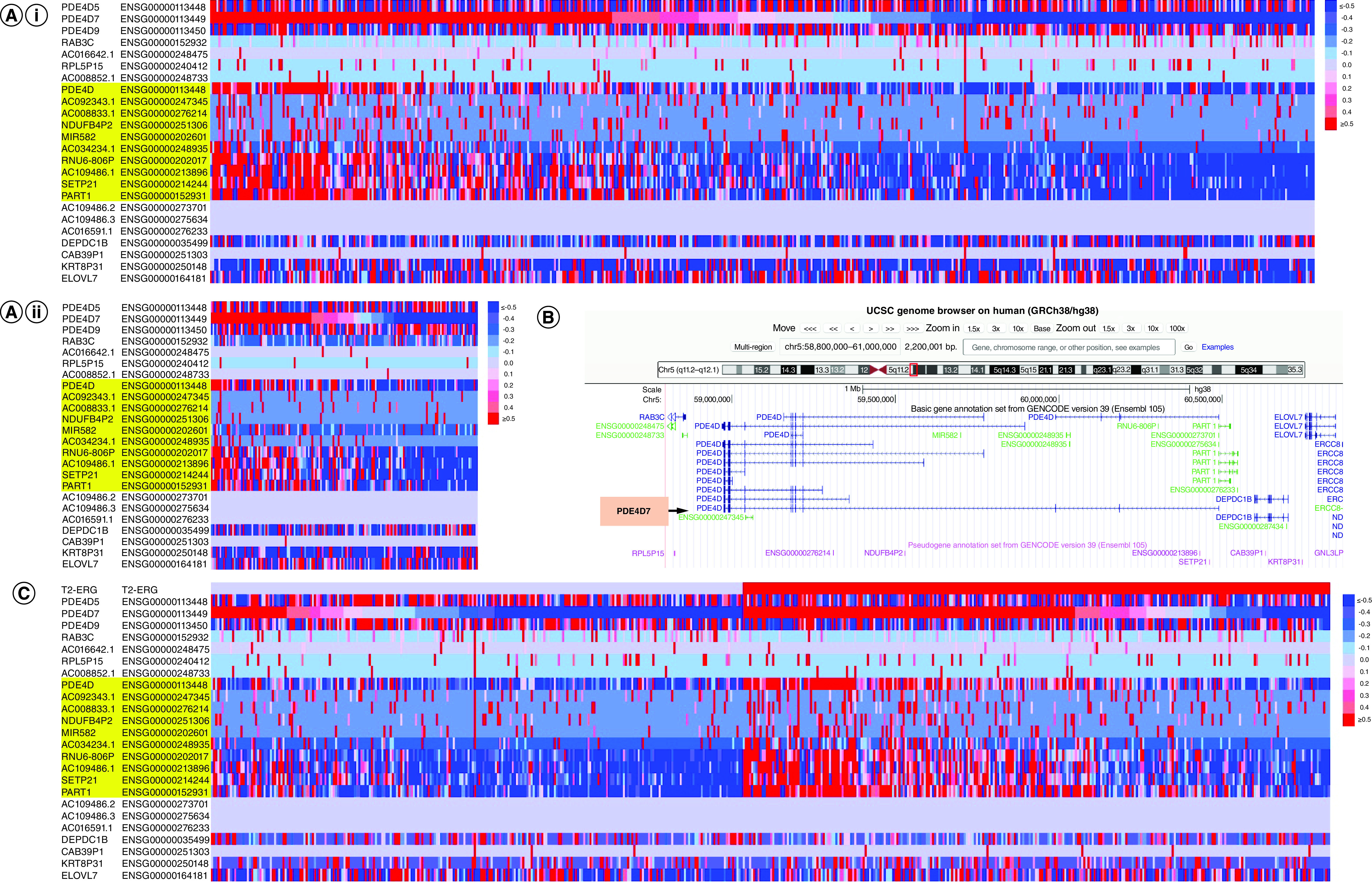
Genome expression on chromosome 5 within *PDE4D* coding region in prostate cancer patients in comparison to the *PDE4D7* score. **(A)** Heat map of gene expression in PCa patient samples from datasets 1 n = 533 **(i)** and 2 n = 151 **(ii)** ordered according to their *PDE4D7* scores from high to low. Gene expression values of the shown transcripts located in the genomic region of the *PDE4D* gene on chromosome 5 were determined by NGS RNA sequencing. Transcript names highlighted in yellow show p-value <0.05 in expression difference between samples with high versus low *PDE4D7* score. **(B)** Chromosome 5 genome alignment in *PDE4D* coding region between approx. 59,000,000–60,500,000 bp from UCSC genome browser (GRCh38/hg38 Human Dec 2013 assembly), accessed on 14 April 2022. *PDE4D7* isoform annotated by arrow and orange text box. **(C)** Heat map of gene expression in PCa patient samples from datasets 1 (n = 533) ordered according to their *TMPRSS2-ERG* (T2-ERG) fusion status.

Our analysis of the data suggests a distinct correlation between *PDE4D7* expression and the expression of a subset of lincRNAs, miRNAs and pseudogenes located between chr5:60,000,000 to chr5:60,500,000 [Figure 1B]. Importantly, all trends observed are correlated between both independent datasets [Figures 1A (i) & (ii)].

Specifically, RNA transcripts located within the first 3 exons of *PDE4D7* are coincidentally downregulated, with *AC034234.1*, *RNU6-806P*, *AC109486.1*, *SETP21*, *PART1* and the entire *PDE4D* gene exhibiting a similar overall expression pattern to *PDE4D7* among samples. These genes showed a p-value < 0.05 in difference of expression between samples with high versus low *PDE4D7* scores [Figures 1A (i) & (ii)], alongside *AC092343.1*, *AC008833.1*, *NDUFB4P2* and *MIR582*. While *AC109486.1* exhibits the similarity in expression, *AC109486.2* and *AC109486.3* show no expression in any sample, alongside *AC016591.1*. Interestingly, all samples with no expression are found in the region overlapping *PART1* which exhibits similar expression to *PDE4D7*, revealing that this entire region is not deleted.

*CAB39P1* showed mostly lack of expression across all samples, however a subset of samples exhibited increased expression irrelevant to *PDE4D7* level. *AC016642.1, RAB3C*, *RPL5P15* and *AC008852.1* revealed similar lack of expression to *CAB39P1* however with more anomalous samples showing altered expression. The most varied expression profiles were *DEPDC1B, KRT8P31* and *ELOVL7*, as well as *PDE4D5* and *PDE4D9*, reflecting no obvious correlation to *PDE4D7*. Of note is that *TMPRSS2-ERG* fusion negative (*TMPRSS2-ERG-*) samples show more pronounced downregulation of genomic elements than *TMPRSS2-ERG* fusion positive (*TMPRSS2-ERG+*) samples, similar as observed relative to *PDE4D7* score [Figure 1C].

## Discussion

Our data shows that *PDE4D* is the only protein coding gene exhibiting an identical expression pattern to *PDE4D7*. Böttcher *et al.* (2016) noted that long *PDE4D* isoforms are significantly downregulated during PCa progression. However, while *PDE4D5* and *PDE4D9* are known to be downregulated in both localized AS PCa and CRPC tissue, *PDE4D7* is only diminished in CRPC, rendering it a more specific measure of disease progression [6].

Other RNAs mapped to the *PDE4D* coding region exhibiting PDE4D7-like downregulation are pseudogenes (*SETP21, RNU6-806P*), nucleotide sequences (*AC034234.1* and *AC109486.1*) and long non-coding (lnc) RNAs (*PART1*). Aside from *PART1*, no function has yet been ascribed to these genes, however *PART1* has been extensively researched with regards to cancer development and progression. *PART1* is predominantly expressed in the prostate, relies on androgens for transcriptional regulation in PCa and is upregulated in PCa tissue [10,11]. Interestingly, while *PART1* is regulated via androgen signaling, *PDE4D7* transcription is not, despite the *PDE4D7* 5′UTR coding region overlapping with antisense *PART1* [4]. Henderson *et al.* (2014) reported that *PART1* and *PDE4D7* exhibit positively correlated mRNA expression within PCa cell lines and xenografts, which our results further confirm [4]. However, we are the first to report the similarity in expression among various genes located within the *PDE4D7* coding region of Chr5q12. Given the lack of functional characterization of many of these genes, and their correlation with *PDE4D7* expression in PCa, further research is warranted to determine their functional significance in PCa progression.

*miR-582*, which is implicated in PCa metastasis, correlated with *PDE4D7* expression however the association was less robust than that of *PART1*. Similar to *PDE4D7*, downregulated expression of *miR-582-3p* and *miR-582-5p* is associated with an advanced PCa phenotype, with diminished expression observed in bone metastatic PCa tissue, correlating with reduced bone metastasis-free survival [12]. It is notable that although *miR-582* expression correlated with *PDE4D7* pattern, there is high variability among samples, with many exhibiting no expression at all.

Correlation between *PDE4D7* level and *TMPRSS2-ERG* fusion status has previously been identified, with upregulated *PDE4D7* expression in positive *TMPRSS2-ERG* tumors (*TMPRSS2-ERG+*) and low-grade PCa phenotype [7]. This gene rearrangement is evident in approximately 50% PCa patients, leading to overexpression of the oncogene *ERG* and subsequently enhanced proliferation and many hallmarks of cancer [13,14]. Given that the *PDE4D7* gene contains an ERG binding site, it was speculated that *PDE4D7* may be regulated via *ERG* transcription [7]. Our data further supports this theory, with negative *TMPRSS2-ERG* tumor samples (*TMPRSS2-ERG-*) exhibiting downregulation of genes akin to those identified upon stratification by *PDE4D7* score.

## Conclusion

In summation, our data identifies the downregulation of an entire chromosomal region of Chr5q12 mapping to the *PDE4D7* domain throughout PCa progression. This pinpoints not only driver genes but also passenger genes which may be a fundamental feature of carcinogenesis. *PDE4D7* and *PART1* have previously been proposed as separate biomarkers for PCa [1,11], however measuring these two genes in combination, or alongside others identified here with correlative expression, could further enhance the specificity of these biomarkers in prognostic approaches. In this era of precision medicine, biomarkers that can be measured to reflect an accurate state of disease, or are implicated in disease pathology, are vital for improving diagnostics and therapeutics.

**Summary points**

- *PDE4D7* represents a promising biomarker for PCa progression, with inverse expression correlating with disease aggression.
- Next generation RNA sequencing identified many additional genetic sequences located in the *PDE4D* coding region with similar expression patterns to *PDE4D7* in PCa tissue samples.
- Many of the biological functions of the *PDE4D7*-associated genes are currently uncharacterized, with these findings suggesting their involvement in PCa.
- These newly identified genes with correlating expression to *PDE4D7* could be used as biomarkers to enhance prognostics when measured alongside *PDE4D7*.
